# Dialysis Disequilibrium Syndrome in a Patient With Acute Kidney Injury on Chronic Kidney Disease

**DOI:** 10.7759/cureus.15608

**Published:** 2021-06-12

**Authors:** Hajime Sanada, Kaori Yamaguchi, Taito Miyake

**Affiliations:** 1 Division of Nephrology and Rheumatology, Kouseiren Takaoka Hospital, Takaoka, JPN

**Keywords:** hemodialysis, disequilibrium syndrome, chronic kidney disease, reverse urea effect, cerebral edema

## Abstract

Dialysis disequilibrium syndrome (DDS) is a neurological complication that has been known to occur after hemodialysis (HD). In recent years, the prevalence of DDS has been low as the symptoms are widely recognized; hence, preventive therapies, such as the slow and gentle procedure for HD, are often administered before starting dialysis. However, once DDS occurs, it may cause seizures, coma, and even death in severe cases. Since there has been no established treatment, recognizing risk factors and preventing the syndrome is important.

A 76-year-old man was admitted to our hospital due to exacerbation of chronic heart failure. He also had a history of chronic kidney disease and had consulted with his home doctor about the preparation for HD a month before admission. After treatment with diuretics, the symptoms ameliorated, but he experienced presyncope and malaise. Laboratory tests revealed acute anemia and a decrease in renal function. Upper gastrointestinal endoscopy revealed active bleeding from a gastric ulcer, which was successfully stopped. However, his consciousness deteriorated because of uremia; hence, HD was initiated. We used a cellulose triacetate membrane with a surface area of 1.3 m^2^ and maintained a dialysate flow rate of 500 ml/min with a blood flow rate of 120 ml/min. Four hours after starting HD, he suddenly developed generalized tonic convulsions. The dialysis was immediately stopped, and the patient was transferred to an intensive care unit. A computed tomography scan of the head showed mild edematous change of the brain, and laboratory tests also revealed a rapid decrease of urea nitrogen. We rationalized that he might have developed DDS. After injection of levetiracetam for the treatment of seizures, we initiated continuous hemodiafiltration as renal replacement therapy. Fortunately, his consciousness gradually improved, and he was completely alert on day 18 after admission.

With reference to our current report, DDS can occur even following acute kidney injury, as the progression rate of the injury and accumulation of blood urea may not correlate with the risk of the syndrome.

## Introduction

Dialysis disequilibrium syndrome (DDS) was first reported by Kennedy et al. in 1962 [1]. It is an acute neurological complication that occurs during or after hemodialysis (HD). Mild symptoms include fatigue, headache, nausea, vomiting, muscle cramps, and tremors. However, it can also result in coma, seizures, and even death in severe cases [2].

DDS is caused by cerebral edema developed due to an osmotic gradient that is formed between the brain and plasma by rapid HD. The main cause of the gradient is a difference in urea clearance between the brain and plasma during dialysis. Urea concentrations in the brain and plasma are nearly the same during homeostasis; however, during HD, the removal of urea from the brain is much slower than that from the plasma [3].

It has been speculated that the incidence of DDS has decreased recently because of its well-known etiology and preventive management. For example, patients at risk, such as those undergoing HD for the first time, are prepared with a slow and gentle procedure to reduce the formation of an osmotic gradient. However, critical cases of DDS are still reported today; therefore, attention should be paid to this syndrome.

Herein, we report a case of DDS that occurred following an acute kidney injury on chronic kidney disease (CKD). The urea concentration in the patient before the occurrence of the syndrome had been followed closely.

## Case presentation

A 76-year-old man was admitted to our hospital because of congestive heart failure. He had a history of CKD, for which he had undergone treatment for more than seven years. Furthermore, the patient also had hypertension for 26 years, but his blood pressure was poorly controlled. He had a history of heart failure caused by CKD and was treated with diuretics. The patient had already consulted with his home doctor about the preparation for HD a month before the current admission. He also experienced dyspnea and palpitations for a couple of days. On admission, the patient had a good appetite and was completely alert, with no neurological symptoms. His height was 168.5 cm, and his body weight was 83.9 kg (his average body weight was 80.0 kg). The patient’s blood pressure was 140/113 mmHg, body temperature was 36.2⁰C, and pulse rate was 107 bpm. His respiratory rate was 20 breaths/min with a SpO2 of 99%, and he felt dyspnea while talking. He did not have any peripheral edema, while the heart and respiratory sounds were normal. The results of the laboratory tests were as follows (Table 1): white-cell count: 11,200/µL, hemoglobin level: 11.8 g/dL, platelet count: 200,000/µL, urea nitrogen level: 83.2 mg/dL, creatinine level: 5.11 mg/dL, and uric acid concentration: 7.0 mg/dL. He had a severe chronic renal failure that might have been caused by nephrosclerosis due to a long history of poorly controlled hypertension. The patient did not undergo renal biopsy because his kidney had already atrophied, but no physical findings or laboratory data indicated any other renal disease. Moreover, laboratory data and the clinical course also revealed that he had anemia from chronic kidney disease and chronic liver dysfunction induced by alcohol.

**Table 1:** Laboratory findings on admission Abbreviations: MCV, mean corpuscular volume; AST, aspartate transaminase; ALT, alanine transaminase; LDH, lactate dehydrogenase; γ-GTP, gamma-glutamyl transpeptidase; ALP, alkaline phosphatase; HbA1c, glycated hemoglobin; BNP, brain natriuretic peptide; PT, prothrombin time; INR, international normalized ratio; APTT, activated partial thromboplastin time

Variable	Reference range	Test results
Hemoglobin (g/dL)	13.7-16.8	11.8
Hematocrit (%)	40.7-50.1	35.5
MCV (fL)	83.6-98.2	96.5
White-cell count (per μL)	3300-8600	11,200
Platelet count (per μL)	158,000-348,000	200,000
Sodium (mEq/L)	138-145	143
Potassium (mEq/L)	3.6-4.8	4.2
Chloride (mEq/L)	101-108	117
Calcium (mg/dL)	8.8-10.1	9.2
Phosphorus (mg/dL)	2.7-4.6	3.7
Urea nitrogen (mg/dL)	8.0-20.0	83.2
Creatinine (mg/dL)	0.65-1.07	5.11
Uric acid (mg/dL)	3.7-7.8	7.0
Total protein (g/dL)	6.6-8.1	5.8
Albumin (g/dL)	4.1-5.1	3.5
AST (U/L)	13-30	70
ALT (U/L)	10-42	98
LDH (U/L)	124-222	286
γ-GTP (U/L)	13-64	124
ALP (U/L)	38-113	82
Glucose (mg/dL)	73-109	98
HbA1c (%)	4.9-6.0	5.2
BNP (pg/mL)	<18.4	846.9
PT (%)	70.0<	133.4
INR	<1.20	0.84
APTT (seconds)	0.0-40.0	24.3
fibrinogen (mg/dL)	150-450	249

Chest radiograph showed a cardiothoracic ratio of 57.7%, and costophrenic angles were bilaterally dull. An electrocardiogram revealed atrial fibrillation, which had never been detected before. It also revealed an ejection fraction of 66% with no wall motion disorder or severe valvular disease. The left ventricle and atrium were dilated, as well as the inferior vena cava without respiratory changes. On day one, he was treated with intravenous furosemide and oral tolvaptan for volume overload, as well as oral warfarin for atrial fibrillation. The patient showed a quick response to diuretics, with a decrease in body weight to 80.0 kg and resolution of dyspnea on day three. However, on day 10, he complained of malaise and presyncope while standing. On day 12, laboratory tests revealed an acute drop in hemoglobin, a coagulation disorder, and a worsened renal function (Table 2).

**Table 2:** Trends of laboratory findings Abbreviation: INR, international normalized ratio; HD, hemodialysis

Variable	Day 1	Day 9	Day 12	Day 13 before HD	Day 13 after HD
Urea nitrogen (mg/dL)	83.2	119.5	221	224	130.7
Creatinine (mg/dL)	5.11	6.07	8.34	9.91	5.73
Hemoglobin (g/dL)	11.8	11.8	6.2	8.1	9.5
Hematocrit (%)	35.5	34.8	18.1	22.6	25.8
INR	0.84	2.22	5.95	1.11	unmeasured
Sodium (mEq/L)	143	142	141	142	141
Potassium (mEq/L)	4.2	3.1	3.2	3.7	3.2
Chloride (mEq/L)	117	106	107	105	106

Vitamin K was administered for increased international normalized ratio (INR) using warfarin, and red blood cells were transfused. Upper gastrointestinal endoscopy revealed active bleeding from a gastric ulcer, which was successfully stopped. Unfortunately, the bleeding led to renal dysfunction, profound urea nitrogen accumulation, and a rapidly deteriorating consciousness (Glasgow Coma Scale {GCS} E3V4M6). Therefore, an urgent HD was initiated on day 13.

**Figure 1:**
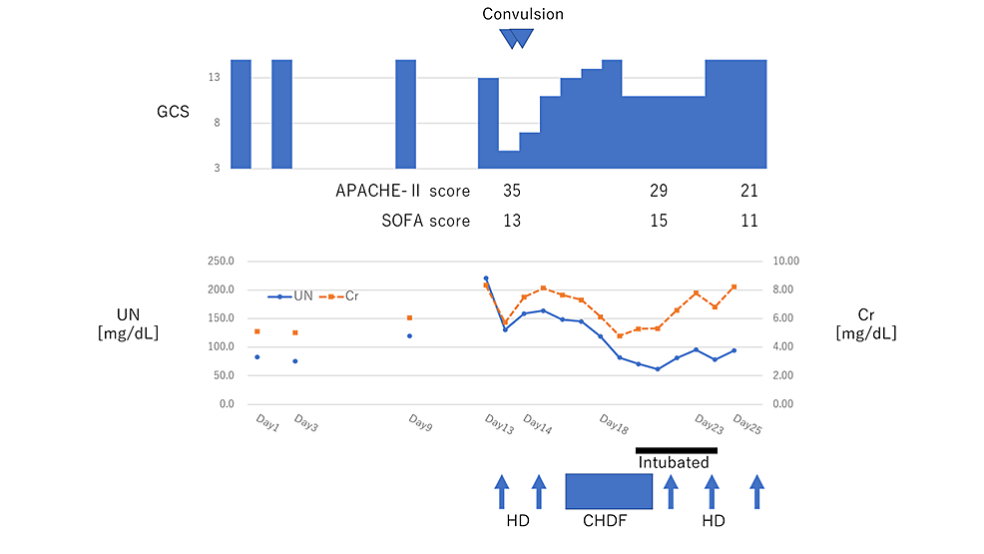
Change in symptoms and laboratory findings Before the first hemodialysis (HD), his Glasgow Coma Scale (GCS) was at 14 (E4V4M6). Tonic convulsions occurred during the first HD, and he subsequently went into a coma (E1V1M3). On the next day, the procedure was repeated but resulted in more seizures. The procedure was subsequently stopped, and the patient was started on continuous hemodiafiltration (CHDF) on day 15. His consciousness gradually ameliorated, and plasma urea concentration also slowly decreased. On day 18, he became completely alert. Subsequently, he was intubated due to severe aspiration pneumonia and was successfully treated by day 23. Abbreviations: APACHE-II score, acute physiology and chronic health evaluation-II score; SOFA score, sequential organ failure assessment score; UN, urea nitrogen; HD, hemodialysis; CHDF, continuous hemodiafiltration

A right internal jugular vein triple-lumen catheter was placed for HD without any complications. We planned the slow and gentle dialysis prescription such that there was the least reduction of plasma urea. We used a cellulose triacetate membrane with a relatively small surface area of 1.3 m^2^. The dialysate flow rate was maintained at 500 mL/min, with a blood flow rate of 120 mL/min. We originally aimed to conduct a short-duration HD; however, the procedure continued for four hours to accommodate red blood cell transfusion. The patient was drowsy (GCS: E3V4M6) even before HD. Three hours after the induction of the procedure, the patient gradually became unresponsive, and he experienced tremors in his mouth and extremities. Approximately four hours after the start of HD, he developed sudden generalized tonic convulsions. We immediately administered diazepam 5 mg intravenously to stop his seizures and stopped the dialysis. The patient subsequently went into a coma (GCS: E1V1M3). Head computed tomography (CT) scans showed a mild edematous change of brain, and laboratory tests revealed a rapid decrease of urea nitrogen (Table 2). We also performed a lumbar puncture, but unfortunately, the cerebrospinal fluid could not be obtained as he had a spinal deformity. As we could not detect any other causes for the adverse symptoms, he was diagnosed with DDS and admitted to the intensive care unit. Levetiracetam (500 mg) was administered intravenously every 12 hours, and no seizures were observed again. Furthermore, glycerol (200 mL) was administered every 12 hours to avoid worsening of DDS. On day 14, we tried to perform the HD procedure again, but tremors were again seen in his extremities 20 minutes after starting the procedure. Accordingly, we stopped, after which his tremors resolved almost spontaneously. The patient then underwent continuous hemodiafiltration (CHDF) on day 15. A smaller polymethyl methacrylate membrane was selected (1.3 m^2^). The blood flow rate was maintained at 100 mL/min, the dialysate flow rate at 500 mL/h, and the filtration flow rate at 500 mL/h. As shown in Figure 1, plasma urea concentrations slowly decreased following the procedure. The patient's consciousness gradually improved, and he was fully alert. However, severe aspiration pneumonia occurred, necessitating intubation and mechanical ventilation (continuous positive airway pressure (CPAP); positive end-expiratory pressure (PEEP): 9mmHg; FiO2: 0.7) on day 18. He was successfully treated with antibiotics, extubated on day 23, and moved to the general ward on day 25.

## Discussion

In the current case report, we considered the risk of DDS to be relatively low before starting the first HD because blood urea nitrogen level increased in a noticeably short period. Our hypothesis might have been incorrect, according to the general etiology of DDS. The syndrome is caused by cerebral edema due to the formation of an osmotic gradient between the brain and plasma, which may occur after an HD. This theory has been confirmed in animal studies [3,4] and has been proven using clinical image analysis. CT scans of the head performed before and after HD revealed that cerebral density significantly decreased after the procedure. This change indicated the progression of cerebral edema [5]. A similar outcome was seen in an MRI study in uremic rats [6].

The main cause of this osmotic gradient is the so-called “reverse urea effect.” Urea is rapidly removed from the plasma during HD, but the reduction from the brain is much slower than from the plasma because urea cannot freely transit across the blood-brain barrier (BBB) [7]. In an animal study, uremic rats that underwent HD had a 53% decrease in plasma urea but only a 13% decrease in urea in the brain [8]. This imbalance could result in an osmotic gradient that causes water to move and accumulate in the brain, resulting in cerebral edema. Thus, it may not be important to determine how fast urea accumulates, but rather attention should be paid to other symptoms of DDS despite the time course of kidney injury and urea nitrogen accumulation.

Animal models of chronic renal failure demonstrate a differential expression of proteins in the brain that transport water and urea. Water crosses cell membranes via aquaporins (AQPs) and urea transporters (UTs). AQP4, AQP9, and UT-B1 are mainly expressed in the brain. It has been confirmed that expression of UT-B1 decreased, while expression of AQP4 and AQP9 increased in the brains of uremic rats [9]. The changes in the expression of these transporter proteins could result in the progression of cerebral edema through HD in chronic renal failure patients, as these findings support the “reverse urea effect” hypothesis.

Moreover, there has been no confirmed treatment for DDS. The administration of osmotically active substances, such as mannitol or glycerol, is often attempted [10]; however, it may not be an effective treatment [11]. Therefore, the prevention of DDS is more important than treatment after its development. Firstly, the risk factors of DDS should be recognized: the date of the first dialysis treatment, patient's age (child and elderly patients), high blood urea concentration, hypernatremia, hyperglycemia, metabolic acidosis, preexisting neurologic abnormalities, and cerebral edema [12]. Secondly, the procedure for HD should be performed slowly and gently. Furthermore, no randomized controlled trials have been attempted to achieve the best HD prescription to avoid DDS. Some experts have reported a 40% reduction in plasma urea in a two-hour HD procedure to be reasonable for preventing DDS [10,11]. In patients with a high risk of DDS, we should consider performing CHDF to prevent DDS. CHDF allows the slow removal of solutes from the plasma; hence, this procedure was prescribed to the current patient. Fortunately, we could avoid the recurrence of DDS, but there has been only one case report of its occurrence during CHDF [13]. Since DDS has been tough to avoid altogether, special attention should be paid to high-risk patients during HD, and HD should be stopped immediately when signs of DDS, such as tremors or alteration of consciousness, are seen.

The prognosis of DDS yet remains unclear. Many fatal cases of DDS have been previously reported [2,14-17]. However, some patients have fully recovered from DDS, like our patient [18-20]. This suggests that the prognosis may not be very bad if the patient can tolerate the acute phase; therefore, the patients should be monitored carefully to not miss signs of DDS exacerbation for at least a couple of days after starting HD.

## Conclusions

We reported a case of DDS that occurred in a patient with acute kidney injury on chronic kidney disease during the first HD despite a gentle dialysis prescription. The patient was closely monitored for blood urea nitrogen throughout his clinical course. There have been a few reports of DDS in which blood urea nitrogen was closely followed, but there also have been cases of DDS in brain trauma patients or patients who had undergone brain surgeries. They also had the typical risks of DDS, which exacerbated cerebral edema in neuro-intensive care units. Although our patient did not have a structural disorder in his brain, he developed DDS during his first HD with the gentle dialysis prescription. To the best of our knowledge, this is the first case report in which blood urea was closely followed until DDS occurred during a gentle HD procedure. We conclude that more attention should be paid to every patient during their first HD procedure, as the rate of blood urea nitrogen accumulation may not correlate with the occurrence of DDS.
